# Cooccurrence of Multiple Sclerosis and Idiopathic Basal Ganglia Calcification

**DOI:** 10.1155/2015/838243

**Published:** 2015-08-16

**Authors:** M. Abedini, N. Karimi, N. Tabrizi

**Affiliations:** Department of Neurology, Clinical Research Development Unit of Bou Ali Sina Hospital, Mazandaran University of Medical Sciences, Sari 48158 38477, Iran

## Abstract

Multiple sclerosis (MS) is a chronic inflammatory demyelinating and neurodegenerative disease of central nervous system that affects both white and gray matter. Idiopathic calcification of the basal ganglia is a rare neurodegenerative disorder of unknown cause that is characterized by sporadic or familial brain calcification. Concurrence of multiple sclerosis (MS) and idiopathic basal ganglia calcification (Fahr's disease) is very rare event. In this study, we describe a cooccurrence of idiopathic basal ganglia calcification with multiple sclerosis. The association between this disease and MS is unclear and also maybe probably coincidental.

## 1. Introduction

Multiple sclerosis (MS) is a chronic inflammatory demyelinating disease of central nervous system that typically strikes young adults, especially women. The pathobiology of MS includes inflammatory and neurodegenerative mechanisms that affect both white and gray matter [1]. MS has multifactorial etiology, including environmental, immunological, and genetic factors [2]. In general, the exact cause and pathogenesis of MS remain unknown. Several researches reported the coincidence of MS and other neurologic conditions [2–5] but to our knowledge this is probably the first report of cooccurrence MS and idiopathic calcification of the basal ganglia. Idiopathic calcification of the basal ganglia that is also known as Fahr's disease is a rare neurodegenerative disorder of unknown cause with a prevalence of <1/1,000,000 that is characterized by sporadic or familial brain calcification [6, 7]. Basal ganglia calcification can be asymptomatic or can be associated with neuropsychiatric and motor symptoms [8]. Though the usual age of presentation of this disease is 4th-5th decade, it can also be present in childhood or adolescence [9]. The objective of this case report was to describe a young female with MS and Fahr's disease.

## 2. Case Presentation

**Figure 1:**
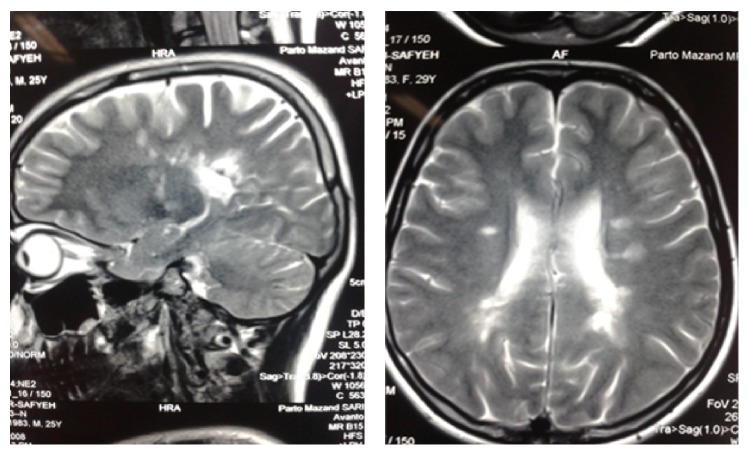
T2 MRI: periventricular plaques.

**Figure 2:**
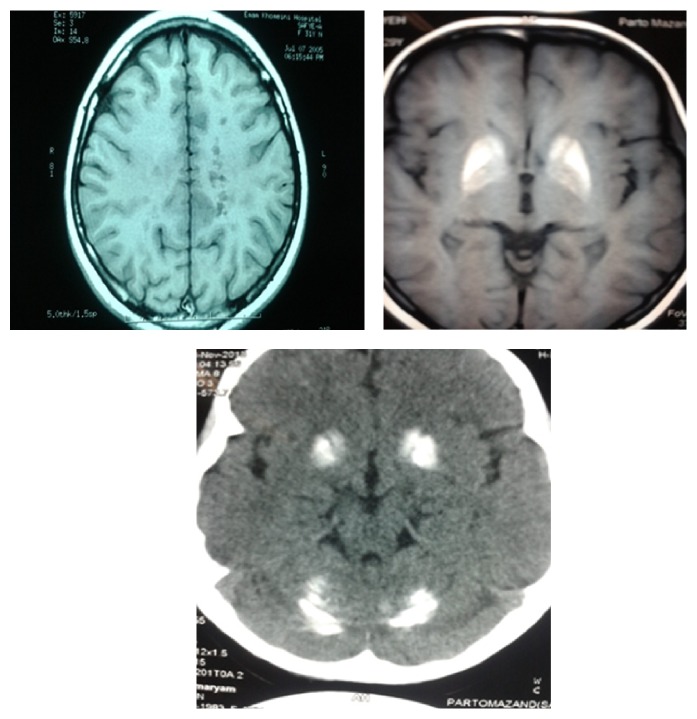
T1 MRI: black hole and basal ganglia calcification at upper side. CT scan: BG and cerebellar calcification at lower side.

We described a 31-year-old Iranian woman with a 9-year history of MS and Fahr's disease. Her complaint had started with fatigability and unsteady gait occasionally, when she was 13 years old. At that time, computerized tomography of brain showed basal ganglion calcification bilaterally. Secondary causes of the brain calcifications were excluded including serum concentration of calcium, phosphorus, parathyroid hormone, thyroid function test, lactate, pyruvate, chemistry profile, sedimentation rate, rheumatoid factor, antinuclear antibodies, ceruloplasmin, and antitoxoplasmosis. The patient had no other signs up to 9 years. At the age of 22, she showed diplopia and paraparesis. Physical examination revealed decreased mood, resent memory disorder, hypertonicity, increased deep tendon reflex, bilateral Babinski, impaired cerebellar tests, and spastic-ataxic gait. The patient did not have a family history and different diagnoses revealed no signs of metabolic or inflammatory etiologies. Magnetic resonance imaging (MRI) of brain and cervical showed multiple areas of increased signal intensity of deep white matter in T2 and FLAIR view, consistent with the diagnosis of MS (Figure 1). The T1 view showed hypersignal intensity at bilateral basal ganglion and cerebellar and also black holes (Figure 2). Computerized tomography (CT) of the brain confirmed bilateral and symmetric calcifications most prominent in the cerebellar hemispheres and basal ganglia (Figure 2). Serum inflammatory markers and autoantibodies were showed negative. Cerebral spinal fluid examination showed increase in immunoglobulin G. With respect to the CT scan findings, clinical history and also normal blood chemistry have diagnosed Fahr's disease and also the findings of brain MRI had confirmed multiple sclerosis. The patient received steroid that vertigo, diplopia, and paraparesis improved up to three months. The patient was treated with interferon *β*. She had an attack that received steroid treatment intravenously. The course of disease was relapsing remitting MS during 8 years. Unfortunately, symptoms of patient progressed and converted to secondary progressive MS from the past year. At present, the patient cannot walk without assistance at a distance of 100 meters.

## 3. Discussion 

MS is a disease that inflammation, demyelination, oligodendrocyte death, gliosis, axonal damage, and neurodegeneration are the main histopathological hallmarks of disease [10] but Fahr's disease is a rare neurodegenerative disorder with characteristic bilateral symmetrical basal ganglia and dentate nucleus calcifications [8]. It is not clear whether the calcification in Fahr's disease is a metastatic deposition of calcium, secondary to local disruption of blood brain barrier, or is due to disorder of neuronal calcium metabolism [11]. The exact pathological process is not fully understood; however, it is thought to be a slowly progressing metabolic or inflammatory process within the brain [12]. The reason of cooccurrence of MS and FD in this patient is unclear. MS may occur independently of FD and the combination of the two diseases in this case was accidental. On the one hand, development of an inflammatory CNS disease in a patient suffering from a primarily neurodegenerative disorder addressed the role of neuronal activity in maintaining immune surveillance and controlling immune functions of the CNS [13]. On the other hand, the injury of neurons and blood brain barrier in the FD caused release of inflammatory mediators in these cells and increased production of proinflammatory cytokines and T-lymphocytes into the brain of lesion. In some studies reported, MS is a primary neurodegenerative disease and the disruption in BBB is likely to be the key pathogenic event in MS and could be triggered by a variety of mechanisms [14].

## 4. Conclusion

It is not clear which pathogenic mechanism plays a role in coexistence of both Fahr's disease and MS. However, when two disease developments, each of unknown reason, occur in the same patient, investigation of their probable association is warranted. Research is recommended about concurrence of MS with brain calcification.
